# A Scientometric Systematic Review of Entrepreneurial Wellbeing Knowledge Production

**DOI:** 10.3389/fpsyg.2021.641465

**Published:** 2021-03-31

**Authors:** Nicolás Contreras-Barraza, Juan Felipe Espinosa-Cristia, Guido Salazar-Sepulveda, Alejandro Vega-Muñoz, Antonio Ariza-Montes

**Affiliations:** ^1^Facultad de Economía y Negocios, Universidad Andres Bello, Viña del Mar, Chile; ^2^Departamento de Ingeniería Industrial – Facultad de Ingeniería, Universidad Católica de la Santísima Concepción, Concepción, Chile; ^3^Public Policy Observatory, Universidad Autónoma de Chile, Santiago, Chile; ^4^Social Matters, Universidad Loyola Andalucía, Córdoba, Spain

**Keywords:** entrepreneurship, satisfaction, happiness, job-satisfaction, mental-health

## Abstract

This article presents a scientometric study regarding entrepreneurship and its relationship with wellbeing. The study presents a systematic review and measures impact and relational character to identify the relevance of countries, research organizations, and authors in the field of entrepreneurial wellbeing. The study poses the following research questions: What is the nature of the evolution of scientific knowledge in the entrepreneurial wellbeing field? What is the nature of the concentration in terms of geographical distribution and co-authorship level of knowledge production in the entrepreneurial wellbeing field? What are the knowledge trends in knowledge production for entrepreneurial wellbeing literature? The contribution of this research is two-fold. First, in terms of methodology, it contributes study into the use of a more robust approach to search for the scientometric trends about entrepreneurship wellbeing in addition to the PRISMA review tools and the PICOS eligibility criteria. Secondly, the study presents research updates in the search for results for the last 2 years of knowledge production. This upgrade is particularly important in a research field that presents exponential growth, where 2019 and 2020 presented almost double the amount of knowledge production compared to 2017 and 2018.

## Introduction

In a much-cited definition of entrepreneurship, Shane and Venkataraman define the entrepreneurship research field as the “scholarly examination of how, by whom, and with what effects opportunities to create future goods and services are discovered, evaluated and exploited” (Shane and Venkataraman, 2000, p. 218). Such a definition of entrepreneurship opens up further possibilities to enlighten us on the subjective and psychological aspects of the entrepreneurship phenomenon. The entrepreneur takes risks, makes decisions, takes advantage of opportunities, and confronts uncertainty. The present study looks to deepen into the subjective and psychological aspects related to entrepreneurship in a growing field of research, that is, the study of wellbeing and entrepreneurship. A research study that investigates the relationship between offerings of recent literature and wellbeing and entrepreneurship could serve to clarify work-life interference aspects of those that embrace entrepreneurial activities.

Wellbeing is a relevant concept for those who produce arrangements to do with work and the economy. For example, The International Labor Organization (ILO) states that wellbeing at the workplace concerns all aspects of professional life. In this sense, the quality and safety of the physical climate, the socio-emotional climate, and work organization are of great importance (International Labor Organization, 2019). One of the cornerstones of professional life is entrepreneurship. Wellbeing at the workplace has been widely studied among employees but much less so in entrepreneurs. The focus on wellbeing has moved to the forefront of scholarly research on entrepreneurship (Shir et al., 2019). In consequence, entrepreneurial wellbeing rapidly becomes a form of access to research job and life satisfaction plus other socio-emotional professional life phenomena.

Following Sánchez-García et al. (2018), the present study's purpose is to organize the growing line of research that connects entrepreneurship and wellbeing, structuring a scientometric analysis of this novel stream of research. The present article contributes by focusing the inquiry on the use of the scientific activity itself and the application of scientometric techniques to measure the impact and relational character to make relevant the countries, research organizations, and authors in the field of entrepreneurial wellbeing. To update some of the results of Sánchez-García et al., this article aims to produce a grounded answer on the subjects of the concentration, actual trends, and nature of the evolution of scientific knowledge of entrepreneurial wellbeing. Following this line of inquiry, the study positions the following research questions, according to the PICOS tool (Methley et al., 2014):

- What is the nature of the evolution of scientific knowledge in the entrepreneurial wellbeing field?
- What is the nature of the concentration in terms of geographical distribution and co-authorship level of knowledge production in the entrepreneurial wellbeing field?
- What are the knowledge trends in knowledge production for entrepreneurial wellbeing literature?

To answer those research questions, authors use a scientometric analytic methodology. According to Kullenberg and Kasperowski (2016), scientometrics meta-analysis examines the production of knowledge, its spatiality, and the relationship between the network of global actors (Moravcsik, 1985; Frenken et al., 2009; Albort-Morant et al., 2017; Vega-Muñoz and Salinas-Galindo, 2017; Mikhaylov et al., 2020). This study focuses on establishing levels of spatial, organizational, and thematic co-authorship using VOSviewer for entrepreneurial wellbeing knowledge production (Van Eck and Waltman, 2010; Köseoglu et al., 2018; Lojo et al., 2019; González-Serrano et al., 2020; Vega-Muñoz et al., 2020). Scientometrics is a study methodology within entrepreneurship studies and has been used previously by Shane and Venkataraman (2000). Busenitz et al. (2003), Cornelius et al. (2006), Qian (2014), Chandra (2018), Sassmannshausen and Volkmann (2018), Duran-Sanchez et al. (2019), Ferreira et al. (2019), and Kang et al. (2019).

This scientometric systematic review contributes to entrepreneurial wellbeing understanding using a dataset built from a JCR-WoS journal collection, as JCR-WoS journals have been defined as the collection with the most significant impact worldwide (Carabantes-Alarcón and Alou-Cervera, 2019; Serrano et al., 2019). Such selection leads to an answer about the concentration, actual trends, and nature of the evolution of scientific knowledge of entrepreneurial wellbeing.

The paper proceeds as follows. First, the study offers a background on entrepreneurship and wellbeing. This background intends to offer a short literature review that brings context to the scientometrics analysis of the field. Later, the article presents the scientometrics methodology and then shows results; later, a discussion for entrepreneurial wellbeing looks at a Scientometric Systematic Review and also discusses the concluding remarks and limitations of this study.

## Research Background

Since the classification of Cornelius et al. (2006), entrepreneurship studies have been concentrated on three lines of research: business management, business history, and economic policy. This article is a systematic review of a business management line but also has a focus on individuals, particularly studying the individual entrepreneur and their behavior, mental processes, satisfaction, mental health, and stress among other personal issues. That is why, in this section, the article elaborates on an updated review of the literature that intends to contextualize the scientometric analysis of wellbeing and entrepreneurship. Firstly, the authors develop the concept of job satisfaction and wellbeing. Later, the text offers an actual view of the relationship between entrepreneurship and self-efficacy. Afterward, the study presents relations between entrepreneurship and health. Then, the text developed de relationship between entrepreneurship and happiness. Finally, the authors set up a revision of literature about entrepreneurship and life satisfaction. But first, this study confronts the more general inquiry about the relationship between wellbeing and then wellbeing and entrepreneurship.

As Wiklund et al. (2019) explain in their review about wellbeing and entrepreneurship literature, it is not easy to define and measure wellbeing. Wellbeing measures and studies can lead to a better understanding of people's quality of life (Stiglitz et al., 2009). The need to understand more about individuals' quality of life had triggered the development of a variety of measurement instruments. For example, Linton et al. (2016) describe 99 different measures for estimating wellbeing. These authors visualize that measures of wellbeing present a significant range that goes from subjective and psychological measures through to objective physical health measurements.

Wellbeing is a broad construct that is both complex and multidimensional (Shir et al., 2019). Wellbeing is a function of subjective and objective influences in people's life experience (Wiklund et al., 2019). Theoretically and empirically, wellbeing offers a variety of avenues regarding their emphasis on external and internal individual conditions. Those differences depend on the outside assessment of external and internal conditions by those that interact with us. Furthermore, differences in the wellbeing conditions also depend on internal evaluation by any person, the objectivity of measurements that researchers construct, and subjective evaluations within the instrument respondents (Shir et al., 2019). More precisely, psychology researchers define wellbeing in terms of subjective wellbeing (SWB), that is, the overall internal state of mental wellness, which does or does not includes pleasure accomplishment and pain avoidance. Subjective wellbeing is what some researchers call hedonic or desire-based wellbeing (Gurin et al., 1960; Bradburn, 1969; Diener, 1984; Diener et al., 1999; Kahneman et al., 1999). On the other hand, other psychologists stress intensity, purpose, and self-realization wellbeing aspects. Such self-realization is known as eudaimonic wellbeing (Ryff, 1989; Deci et al., 2001; Keyes, 2006; Diener et al., 2010).

An important aspect to consider about the theoretical construct of wellbeing is the predictive role, emphasizing the importance of the contextual, intrapersonal, and dynamic contribution of intrapersonal and contextual factors (Diener, 2000; Damsbo et al., 2019; Santini et al., 2020). From the contextual perspective, there is the major influence of external life circumstances like material conditions; life events; and sociopolitical contexts on the subjective experience of individuals (Galinha and Pais-Ribeiro, 2011). According to this perspective, adverse circumstances affect WB (Feist et al., 1995). In the case of intrapersonal factors, results indicated that intrapersonal variables are stronger determinants of SWB than contextual factors (Diener and Ryan, 2009; Leite et al., 2019). This perspective received empirical support, placing personality and positive predisposition as the main predictors of WB and SWB (Lucas, 2008; Zhang et al., 2019). The integrative perspective defends how WB and SWB are influenced by multiple variables, like the individual's emotional state, past events, expectations of the future, and social comparisons, like in a dynamic interaction (Suh et al., 1998; Schwarz and Strack, 1999). In this perspective, the main objectives of studies consist of understanding the psychological processes inherent to the different measures of WB and SWB (Diener and Biswas-Diener, 2000; Galinha and Pais-Ribeiro, 2011).

Researchers have seen entrepreneurship as a process phenomenon where actors enmesh goals, desires, and hopes with their actions in the world. Consequently, entrepreneurship may facilitate the fulfillment of a person's fundamental psychological requirements and, at the same time, be a critical aspect that affects psychological wellbeing (Williams and Shepherd, 2016; Shepherd and Patzelt, 2017; Shir et al., 2019). Wiklund et al. (2019, p. 582) define entrepreneurial wellbeing as “the experience of satisfaction, positive affect, infrequent negative affect, and psychological functioning in relation to developing, starting, growing, and running an entrepreneurial venture.” This interesting relationship between entrepreneurship and wellbeing will be further expanded upon in the next subsections where we elaborate on several aspects of entrepreneurship and wellbeing.

### Entrepreneurship and Job Satisfaction

Jensen et al. (2017) claim that entrepreneurs' activities may bring economic and non-economic benefits. Authors express that wellbeing could be of high importance to those non-economic gains. For a Chinese sample of 33,519 entrepreneurs, Jensen and his colleagues demonstrated that innovation activities related to entrepreneurship may have a positive effect on an individual's job satisfaction, the balance between work and family, and general life comfort. Furthermore, several recent studies indicate a greater interest in the psychological results of entrepreneurial efforts, such as psychological wellbeing (Uy et al., 2013; Houshmand et al., 2017; Hahn, 2019), quality of life (Tobias et al., 2013; Reuschke, 2019), job satisfaction (Millán et al., 2013; Soboleva, 2019), and business satisfaction (Carree and Verheul, 2012). Examining such psychological outcomes and their antecedents is important because life satisfaction is associated with many outcomes in people's lives, including health, personal income, longevity, citizenship, and social relationships (Diener et al., 2015). Studies have also revealed positive effects of individual happiness and job satisfaction on various aspects of individual job performance (Cropanzano and Wright, 2001), work unit performance (Harter et al., 2003), and business performance (Van De Voorde et al., 2012; Wood et al., 2012). These findings intensify the economic interest of policymakers around the world to explore the history of business life satisfaction as a potential engine of economic growth; besides Naudé et al. (2013), we found that opportunity-motivated entrepreneurship may contribute to a nation's happiness but only to a certain point, at which the effects of happiness begin to decline. Moreover, our results suggest that a nation's happiness affects early-stage opportunity-driven entrepreneurial activity.

Academic literature demonstrates that self-employed persons enjoy greater autonomy than non-self-employed individuals (Lange, 2012). Furthermore, those self-employment persons experience a higher level of job involvement and job satisfaction than those employed in organizations.

Nevertheless, self-employment persons also feel higher levels of work–family conflict and lower family satisfaction (Parasuraman and Simmers, 2001). In consequence, there is a tradeoff between job and family satisfaction, and this fact can negatively impact the level of entrepreneurs' wellbeing.

### Entrepreneurship and Self-Efficacy

Researchers in entrepreneurial studies are increasingly interested in the psychological wellbeing of entrepreneurs (Ryff, 2018; Wach et al., 2020). One of these psychological wellbeing studies about entrepreneurship had its origins in Bandura (1977). Bandura defined self-efficacy as the belief in the ability to control and positively significantly affect life. Various studies indicate that having a high degree of self-efficacy has a significant impact on the positive and happy state of a person (see also Caprara et al., 2006). Zhao et al. (2020) indicated that entrepreneurial decision-making and entrepreneurial experience affect household happiness significantly. Family wellbeing is significantly increased if the family is entrepreneurial, and it will be higher if the family is actively entrepreneurial. Both entrepreneurial experience and entrepreneurial investment of time have a significantly positive effect on the probability of family wellbeing.

Self-efficacy in entrepreneurship is defined as the belief that an individual has the ability to fulfill the essential roles and associated tasks with the entrepreneurship processes. Those essential roles are, for example (Fordyce, 1988), identification and commercialization of new products and services (McGee et al., 2009). Furthermore, Marshall et al. (2020) claim that accessibility of resources leads to entrepreneurial wellbeing through an entrepreneurial self-efficacy mechanism.

Additionally, studies agree that entrepreneurs with higher self-efficacy are likely to develop strong business identities, which are critical to the successful growth of a new company (Brändle et al., 2018). Strong business identities allow for behaviors with indications of high self-efficacy where entrepreneurs can feel safe in their new businesses and, therefore, increases their prediction improvements probability (Stroe et al., 2018). Clearer goals and plans, along with greater confidence, lead to successfully executing plans. Those plans will result in a greater sense of happiness and satisfaction for entrepreneurs. Self-efficacy has also been considered an essential mediator in various aspects of wellbeing and desired attitudes in entrepreneurs and also in behaviors related to the leadership necessary to carry out entrepreneurial activities (Nielsen and Munir, 2009; Nielsen et al., 2009; Liu et al., 2010). For Dijkhuizen et al. (2018), the importance of entrepreneurs' wellbeing is that it is a key factor in long-term subjective financial and personal entrepreneurial success. The practical implication is that entrepreneurs should maintain and improve their own wellbeing to achieve positive long-term business outcomes.

### Entrepreneurship and Health

There is some research on entrepreneurship that explores the topic of health, e.g., working on how a business career impacts psychology (Tetrick et al., 2000; see Kets De Vries, 1977) and physics (Boyd and Gumpert, 1983; Buttner, 1992). Further, some recent studies have shown researchers interest in continuing to investigate this phenomenon (Heikkilä et al., 2019; Kearney et al., 2019; Patel et al., 2020). Previous and actual results show that entrepreneurs experience lower overall physical and psychic morbidity. Between other symptoms, there is also lower blood tension and a lower predominance of hypertension. Entrepreneurs also show higher wellbeing and more favorable behavioral wellness signs (Stephan and Roesler, 2010). These authors claim that entrepreneurs experience significantly higher job control and demands compared to employees. Higher job control and demands suggest that entrepreneurs have so-called active jobs and, therefore, can benefit from positive health consequences.

Researchers explain these higher levels of health based on entrepreneur decision power. Indeed, entrepreneurs have a high degree of decision power since they own their company and control work organization and resources like time, money, and asset distribution at their workplace (Rau et al., 2008; Schreibauer et al., 2020). Consequently, research has found that entrepreneurs have higher work control, which leads to a higher level of autonomy and discretion at work, and, therefore, this leads to more opportunities for their skill utilization (Eden, 1975; Lewin-Epstein and Yuchtman-Yaar, 1991; Chay, 1993; Parslow et al., 2004; Stephan et al., 2005; Prottas and Thompson, 2006; Rau et al., 2008; Schreibauer et al., 2020). As a corollary, it is possible to expect that entrepreneurs experience better health compared to employees, as they generally report greater control of work than employees.

### Entrepreneurship and Happiness

The pursuit of happiness and the achievement of wellbeing are two highly debatable concepts that are rife with meanings and nuances that lead to some complexities in the theorizing process, including some cases of overlapping characteristics (Lyubomirsky and Lepper, 1999; Riff and Singer, 2007; Boehm and Lyubomirsky, 2009; Zhao et al., 2020). The concept of happiness can be understood as an individual cognitive representation of the nature and experience of wellbeing (Bojanowska and Zalewska, 2015; Flores-Kanter et al., 2018; Usai et al., 2020). These conceptions can generally be described as the degree to which people emphasize hedonic or eudaimonic dimensions as important aspects for the experience of wellbeing (McMahan and Estes, 2011; Chang and Chen, 2020), bringing the concept closer to the subjective wellbeing of the individual than to your psychological wellbeing. In the literature, in addition to being related to subjective wellbeing (Diener et al., 2006; Hill and Buss, 2008), it is interpreted as emotional wellbeing, positive affect (Fordyce, 1988), and quality of life (Shin and Johnson, 1978; Diener, 2000; Ratzlaff et al., 2000), which suggests that the meanings of happiness may depend on the context and individual emotionality (Diener et al., 2006; Carlquist et al., 2016). These definitions indicate a close relationship between the constructs of happiness, subjective wellbeing, quality of life, and life satisfaction. The relationship between happiness, wellbeing, and work has been validated in numerous studies (Rodríguez-Muñoz and Sanz-Vergel, 2013; Pryce-Jones and Lindsay, 2014; Marques, 2017).

From this base, the relationship between happiness and entrepreneurs is more frequently concentrated on the empirical studies carried out in the comparison between the level of happiness of entrepreneurs and employees (Benz and Frey, 2008), in the comparison between the level of happiness of the different types of entrepreneurs (Arenius and Minniti, 2005; Carree and Verheul, 2012), in happiness and its relationship with creativity (Chang and Chen, 2020; Usai et al., 2020), between the gaps of aspirations and their result real in entrepreneurship (Stutzer, 2004; Schneck, 2014), and in negative emotions that can develop in a competitive environment (Hill and Buss, 2008). Another line that has also been developed is the one that sees the effect of government quality influence on entrepreneur happiness through influencing the institutional environment (Larsson and Thulin, 2018; Li et al., 2019). Entrepreneurs were found to have a significantly higher mean level of happiness than employees. In the workplace, individuals who experienced personal growth and were able to contribute their ideas tended to be happier, relative to others who perceived themselves to be “restricted” (Mahadea and Ramroop, 2015). The study of Mahadea and Ramroop (2015) also found that, on average, happier people tended to be educated, married with children, and treated fairly at work. But having too many children produced reduced individual happiness,

On the other hand, other studies that seek to understand the entrepreneurial process and its relationship with happiness, such as those by Su et al. (2020), have found findings where entrepreneurs in the process of establishing a company can persist in an uncertain environment, acquiring positive emotions. That is to say, the motivation for the sustainability of entrepreneurship originates from both the emotion of happiness and satisfaction from the very act of undertaking the entrepreneurship process, and emotional return is a performance dimension parallel to economic profitability. This conclusion provides a new perspective to reveal the entrepreneurial motivation of entrepreneurs in highly ambiguous environments.

### Entrepreneurship and Life Satisfaction

Work is an essential facet of human life that contributes a large component to wellbeing through job satisfaction (Wright and Cropanzano, 2000). Entrepreneurs obtain satisfaction from leading an independent lifestyle and “being their own” bosses (Bhuiyan and Ivlevs, 2018; Kibler et al., 2019; Zwan et al., 2020). In this vein, Hundley (2001) and Hahn (2019) find that self-employed people are more satisfied with their work, and this is mainly due to greater autonomy, greater flexibility, the potentiality of their skills, and, to a certain extent, their reliance on job security due to self-management.

Empirical work has shown that employees have lower job satisfaction in large companies compared to small companies (Idson, 1990; Benz and Frey, 2008). In this spirit, studies indicate that this job satisfaction level is closely related to the tasks assigned at work. Job satisfaction is related to work tasks themselves and the ability to use employees' initiative in their practice (Benz and Frey, 2008). However, Noorderhaven et al. (2004) observe that the levels of dissatisfaction with life in society are positively associated with self-employment rates. Nevertheless, job satisfaction is not the only variable that a researcher must study in order to determine an entrepreneur's wellbeing. Researchers need to consider numerous other components, for example, being affected factors that may be complex and those that interact with each other (Binder and Coad, 2012, 2013). Since individuals may be able to compensate for high performance in some domains of life with otherwise low achievements, high job satisfaction may be offset by less satisfaction in terms of the family specifically or social life more generally.

Given the various aspects mentioned, this study seeks to establish, through a systematic review of broad coverage, the set of relationships that in the mainstream literature have been indexed, and with impact calculated in the JCR-WoS, those that have been documented on the simultaneous study of the wellbeing and entrepreneurship, using a database established and analyzed through a scientometric meta-analysis.

## Methods

### Study Design

Academic publications play an effective role in generating changes in the world of knowledge (Missen et al., 2020). In particular, Glänzel and Thijs (2004) and Franceschet and Costantini (2010) highlight the effect of co-authorship of an article as a reason to reveal the importance of a study, and this was observed as the achievement of more citations. More in detail, Glänzel and Thijs (2004) and Franceschet and Costantini (2010) note the article co-authorship as its central drive for its achievement of more citations.

Scientometrics as meta-analysis (Kullenberg and Kasperowski, 2016) focusses on knowledge production, the spatiality of knowledge production, and knowledge relationships between the network of global actors (Moravcsik, 1985; Frenken et al., 2009; Albort-Morant et al., 2017; Vega-Muñoz and Salinas-Galindo, 2017; Mikhaylov et al., 2020). Scientometrics relationally studies knowledge production, moving the author's gaze toward spatial and organizational co-authorship, as well as research field themes. In this text, the authors use the VOSviewer tool (Köseoglu et al., 2018; Lojo et al., 2019; González-Serrano et al., 2020; Vega-Muñoz et al., 2020) to perform a whole set of analysis of scientometric data about entrepreneurship wellbeing literature. Scientometrics allows us to strengthen systematic reviews (Porter et al., 2002), and it has been used recently in the field of Psychology (Caffò et al., 2020; Peng et al., 2020) and Business (Iandolo et al., 2019; Inkizhinov et al., 2021); its incorporation of sequential mixed use with PRISMA has also been addressed previously (Kazerani et al., 2017; Cavinatto et al., 2019; Sott et al., 2020).

### Systematic Review Protocol

In this article, we carry out a scientometric review of the literature on entrepreneurial wellbeing, and it seeks to synthesize this scientific literature. We have used strict control mechanisms, such as the PRISMA method, in order to reduce biases to a minimum (Liberati et al., 2009; Urrútia and Bonfill, 2010) in the process of choosing and discarding articles. In addition, we have relied on a previous protocol of explicit criteria, uniformly applied to all articles, in order to narrow the topic and focus on the objectives set.

### Search Strategy

To perform the analysis, the authors defined the next searching strategy: (TS=(entrepreneur^*^ AND (wellbeing OR wellbeing))). Such we used the search terms “wellbeing” and “entrepreneur.” For the first term, we searched it with and without a space between the two words (wellbeing and wellbeing), and we included the asterisk so that the search engine would find all its possible variations (for example entrepreneur, entrepreneurship, entrepreneurial, derived adjectives, and plural uses) (see Table 1). Eligibility criteria have been developed using the population, interventions, comparators, outcomes, and study designs (PICOS) (Methley et al., 2014), which is detailed in Table 2.

**Table 1:** Phases of a scientometric systematic review.

**Meta-analytical phases**	**Description**
Initial	The search vector determination, articles selection according to the PRISMA method and the PICOS eligibility criteria, and data extraction existing in the WoS database.
Production	A global scientific production growth analysis on Entrepreneurship and Wellbeing in annual article numbers published in journals indexed to the JCR-WoS (SSCI-JCR and SCIE-JCR) and its fitness level in terms of exponential growth, according to Price's Law.
Spatiality	The economic geography analysis of scientific production in response to the question “Where is this knowledge produced?” The data extraction and determination of the countries where the authors' affiliation organizations are located and their global mapping follow.
Relational	Existing relationships based on text data are analyzed using the VOSviewer in various topics: • National co-authorship, where knowledge production analysis is Joint with the author's contribution being affiliated with various countries, visualization through graphs, concentration determination, and relationships at the national level. • Organizational co-authorship, where joint knowledge production analysis Joint with the authors' contribution, which is affiliated with various organizations, visualization through graphs, concentration determination, and relationships at an organizational level. • Related Keyword Plus® (KWP), which determinates a relevant KWP set (OKWP) according to Zipf's Law, including the analysis of their use in the article dataset studied, visualization through graphs, concentration determination, and relationships at a thematic level. • Intermediary organizations clusters, where the intersection analysis between the organizational co-authorship and the use that they are making of the OKWP include the following aspects: the organization's establishment, visualization through graphs, concentrations determination and relationships at an organizational level, and the organization's identification, which is created in the knowledge production structure base as a topic of study worldwide. • Key Terms, where we include establishment through text analysis with VOSviewer, from titles and abstracts articles under study, visualization through temporal graphs, temporal identification of the most widely used terms, and thematic trends identification. Its concentration is established by Zipf's law.

**Table 2:** Eligibility criteria (PICOS).

**PICOS**	**Description**
Population	Entrepreneurs, self-employed, business students, CEOs, small business owners, organizationally employed, young workers, customers.
Interventions	Entrepreneurship, self-employment, entrepreneurial education, first job, receive funding to entrepreneurship, participate in an entrepreneurship support program. purchasing at entrepreneurs.
Comparator	Only at the data and metadata level of the articles: Nationality of authorship, Organizational affiliation of authorship, Keywords plus, Key Terms, Publication year. As concentrations discriminant criterion, Bradford's law on journals, and Zipf's law on keywords and key terms are applied.
Outcomes	Relationship (bidirectional) between entrepreneurship and SWB, with particular emphasis on job satisfaction, self-efficacy, health, happiness, and life satisfaction.
Study designs	All study types will be included: qualitative (interviews, focus groups, ethnography), quantitative (survey dataset, cohort studies, cross-sectional studies), and mixed methods studies.

We understand that many investigations related to the traits and actions of entrepreneurs, such as “self-employment,” “business owner,” “independent worker,” and “organizational employer.” These words were included in our search; however, for purposes of maintaining quality in our study, we only considered peer-reviewed articles and those specifically associated with the concept “wellbeing,” as seen in Table 2.

### Data Sources and Data Extraction

We extracted the dataset for this study from SSCI-JCR and SCIE-JCR, which are the only databases of the main Web of Science collection for which the Impact Factor of the Journal Citation Report (JCR) is calculated (Biglu, 2008; Golubic et al., 2008; Navarrete-Cortés et al., 2010; Ruiz-Pérez and Jiménez-Contreras, 2019), restricting itself to only documents of the type articles (DT), independent of the language of the main text (LA), but using data and metadata in English. We excluded all indices without impact calculation: Arts & Humanities Citation Index (A & HCI), Conference Proceedings Citation Index- Science (CPCI-S), Conference Proceedings Citation Index-Social Science & Humanities (CPCI-SSH), Book Citation Index–Science (BKCI-S), Book Citation Index–Social Sciences & Humanities (BKCI-SSH), and Emerging Sources Citation Index (ESCI). The multiple indexing of journals generates for WoS an intersection with PubMed® declared as metadata in the PM field (see the in the Supplementary Data Set for this article); in addition to this, the journals indexed to both JCR bases have high duplicity with the indexed journals in Scopus, and both interaction percentages are reviewed and presented as a result. The Scopus journals, which do not present double or triple indexing with the SSCI and SCIE bases, have not been considered because “Scopus covers a superior number of journals but with lower impact and limited to recent articles” (Chadegani et al., 2013, p. 24). The dataset was downloaded from the website www.webofknowledge.com of Clarivate on November 13, 2020.

### Data Analysis

The first analytical step is the recognition of a possible incremental evolution of scientific knowledge that justifies the research effort (Dobrov et al., 1979; Price, 1986; Garfield, 1987; Spinak, 1998; Escorsa and Maspons, 2001; Vega-Muñoz and Salinas-Galindo, 2017). The identification of incremental evolution is performed on research documented in the main collection of JCR-WoS journals. The main collection of JCR-WoS journals has been defined as the collection with the most significant impact worldwide (Gavel and Iselid, 2008, Carabantes-Alarcón and Alou-Cervera, 2019; Serrano et al., 2019).

Later, the authors evaluated several concentration elements. First, authors used Bradford's Law at the level of journals to measure the concentration adjustment levels of geometric series order (Bulik, 1978; Morse and Leimkuhler, 1979; Pontigo and Lancaster, 1986; Swokowski, 1988; Kumar, 2014; Shelton, 2020). Looking at the concentration adjustment levels of geometric series order; the authors intended to identify a potential concentration journal hub specialized in entrepreneurial wellbeing (Andrade-Valbuena et al., 2019; Marzi et al., 2020; Vega-Muñoz et al., 2020).

In a subsequent analytic step, the authors used Clarivate analytic Keyword Plus®–KWP. KWP represents metadata for articles in this study dataset. Then, the authors computed Zipf's Law (Zipf, 1932) using the square root of those KWP. That is [square_root (KWP) = n_1_], where n_2_ words are considered with a number of occurrences equal to or greater than the occurrences of n_1_, with n_2_ > = n_1_.

### Assessment of Risk of Bias

This research collected information on entrepreneurs' wellbeing from 331 SSCI+SCIE articles. Quality and academic relevance are the central attributes of publications indexed at the SSCI+SCIE database. Those articles are part of the selected JCR-WoS journals collection. Scholars had claimed that JCR-WoS journals became the collection with the most significant impact worldwide (Carabantes-Alarcón and Alou-Cervera, 2019; Serrano et al., 2019). Consequentially, the very selection of journals indexed in SSCI+SCIE with JCR impact led to increased reliability and control risk bias of the article sample.

To ensure additional quality control of the article selection, authors extracted the information following specific objectives, setting out any particular, or self-interest criteria that may have limited the research and results of this investigation. The authors sorted out discrepancies about any selection in this article with the inclusion of a third author who helped to triangulate any disagreement.

## Results

**Figure 1:**
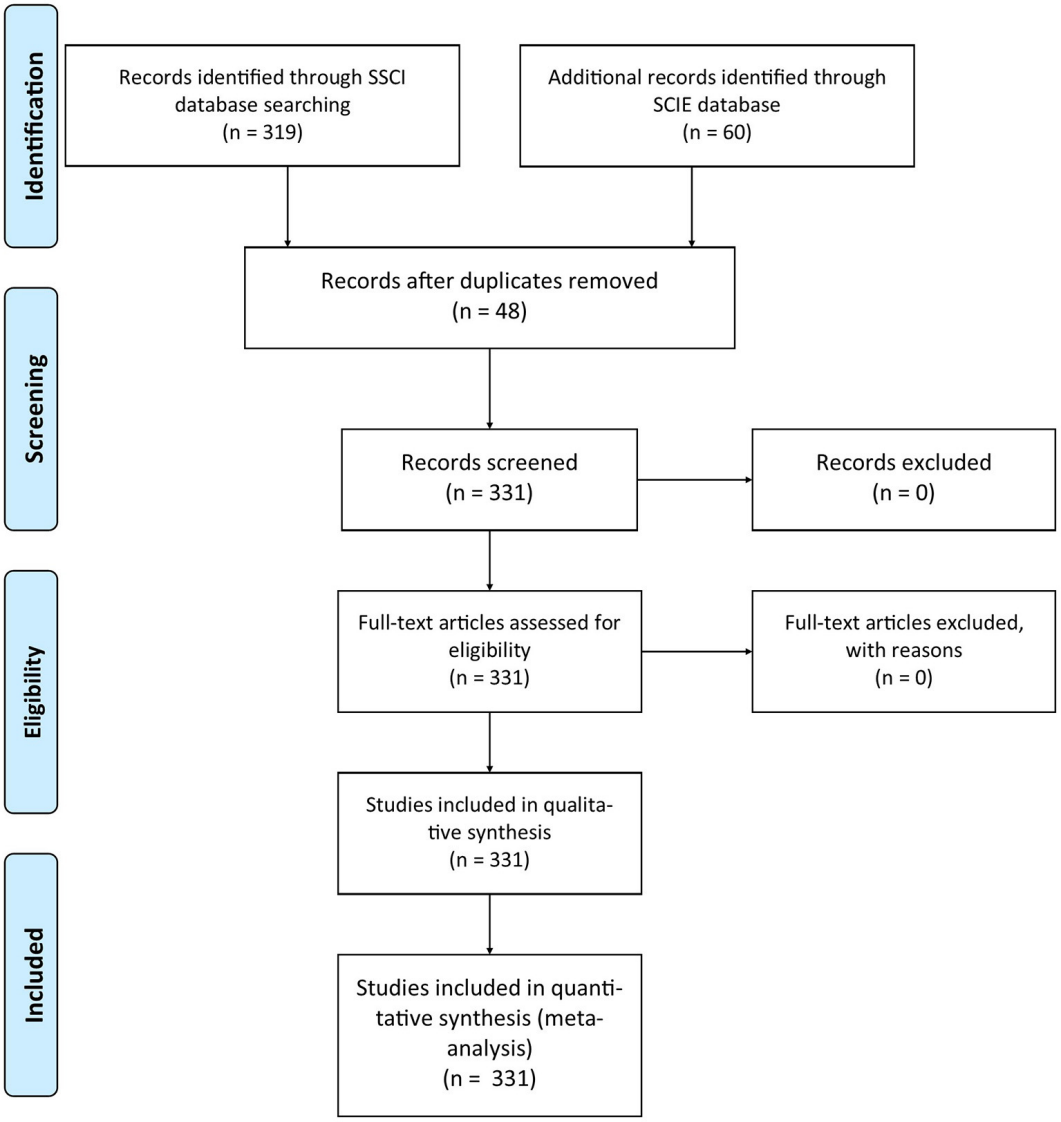
PRISMA flow diagram.

Figure 1 and Table 3 present a flow diagram of the studies from SSCI+SCIE using the systematic procedure explained in the previous method section (Moher et al., 2009).

**Table 3:** Flow diagram of the studies.

**Stage in flow**	**SCIE**	**SSCI**	**Lost**	**Total articles**	**Languages**	**Included articles**
Identification	59	318	0	331	English	320
Screening	59	318	0	331	German	1
Included	59	318	0	331	Norwegian	1
					Russian	6
					Spanish	2
					Swedish	1
					Total articles	331

### Study Selection and Characteristics

#### Synthesized Findings

Between 1995 and 2020, scientists published 331 articles in 222 journals indexed to the SSCI and SCIE at WoS-JCR databases on the topic of entrepreneurship and wellbeing. Journals whose multiple indexing coincides in 100% of cases with journals indexed in Scopus (331 articles) and in 36 cases with journals indexed to PubMed (44 articles, 13%). This number of articles means that scholars publish an average of 13 articles per year. Further, in 2019, a total of 61 works were published, which contrasts with only 1 in 1995. Based on this set, considered as the population of articles under study, the following analyzes were carried out for the samples that are detailed in Table 4.

**Table 4:** Phases, stages, and samples in the scientometric systematic review.

**Meta-analytical phases**	**Stages**	**Sample (*N* = 331)**
Production	World scientific production growth	331 articles (census)
Spatiality	Economic geography analysis of scientific production	331 articles (census)
Relational	National co-authorship (NCA)	331 articles (census) = 50 NCA → 32 NCA – connected
	Organizational co-authorship (OCA)	331 articles (census) = 523 OCA → 141 OCA – connected = 15 OCA – cluster
	Keyword Plus^®^ (KWP)	331 articles (census) = 975 KWP
	Outstanding Keyword Plus^®^ (OKWP), reduction of KWP according Zipf's Law	36 KWP – Outstanding = 36 OKWP = 86 articles
	Intermediary organizations clusters (IOC), by clusters intersection	= 17 articles
	Key Terms (KT) in contemporaneous half-period, and reduction of terms according Zipf's Law	159 articles (2017–2020) = 4,950 terms = 70 KT

**Figure 2:**
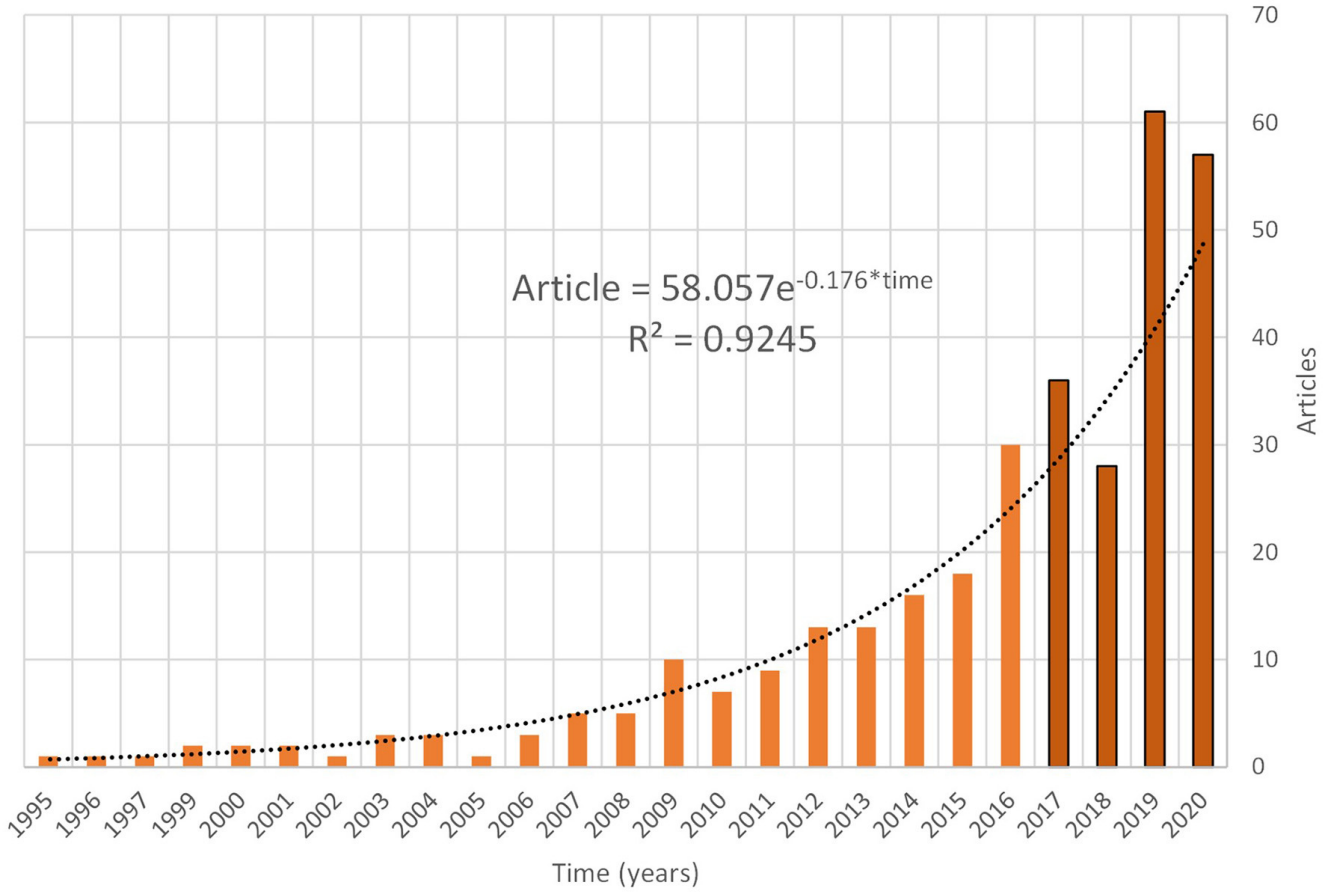
World scientific production growth.

Consequently, the present study highlights an exponential knowledge production growth process in this field of research. Figure 2 presents the aforementioned world scientific production. Such a growth pattern leads us to identify the existence of a worldwide researcher critical mass on the subject. Figure 2 details the current knowledge production of half-periods, represented in dark orange bars, from 2017 to date. This knowledge production curve presents an *R*^2^ of 92% statistical adjustment.

Regarding Bradford's Law, there are no academic journals with a notoriously high concentration of articles. However, despite the lack of homogeneity of the entrepreneurship wellbeing field, it is possible to identify that the growth in knowledge production zones follows a geometric rate with a 0.7% error in the geometric series. This means the geometric series error is not significant (Kumar, 2014). Therefore, the result is statistically consistent. Consequently, the analysis highlights seven journals with participation equal to or >2% in the total world knowledge production: *Journal of Business Venturing* (16 articles, 5%), *Small Business Economics* (12 articles, 4%), *Journal of Business Ethics* (9 articles, 3%), *Sustainability* (7 articles, 2%), *Macromarketing Magazine* (6 articles, 2%), *Theory and Practice of Entrepreneurship* (5 articles, 2%), and *Journal of Happiness Studies* (5 articles, 2%). As a result, although there is no higher concentration in academic journals about entrepreneurship wellbeing research, some academic outlets are beginning to show a preliminary concentration pattern.

**Figure 3:**
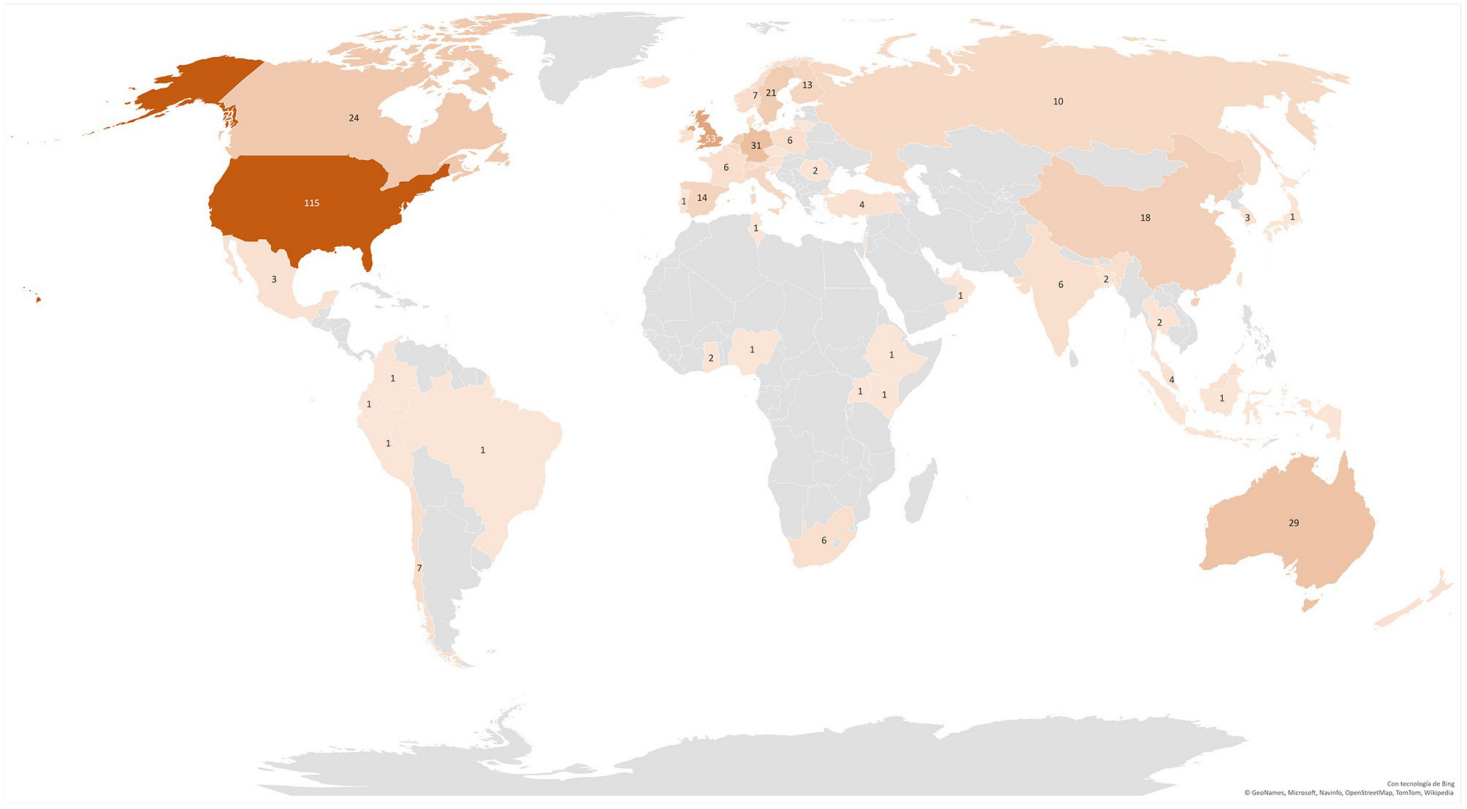
Economic geography of scientific production.

In terms of geographical concentration, the pattern is radically different. Figure 3 represents the world distribution of scientific production in the subject under study, where the participation of 57 countries is identified. Standing out with the highest percentage contribution margins, out of the 331 articles analyzed, are the following: the US with 35%, the United Kingdom with 16%, Germany with 9%, Australia with 9%, and Canada with 7%.

**Figure 4:**
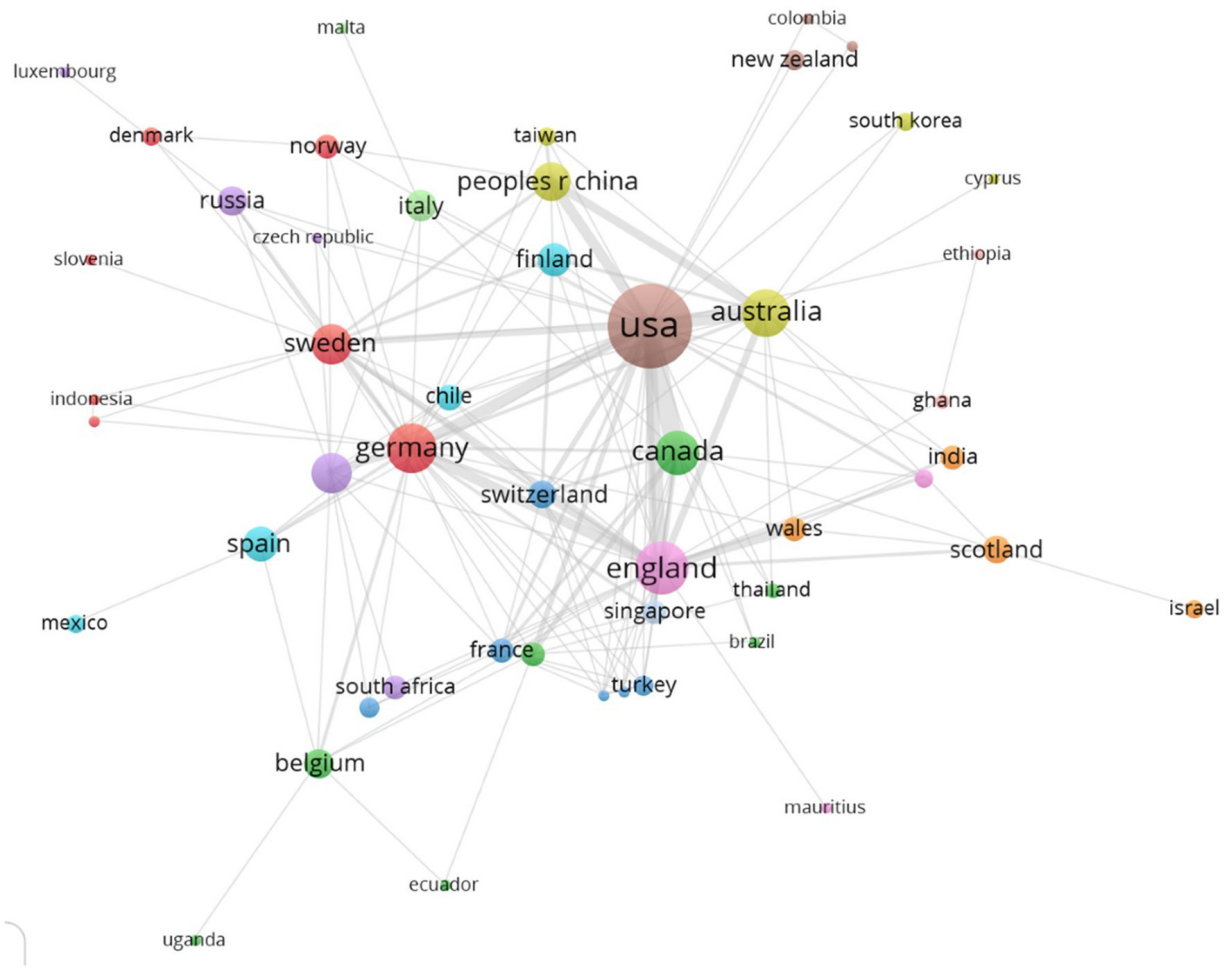
National co-authorship.

Figure 4 complements the above by consistently connecting 50 countries through the VOSviewer software. The United States not only presents notorious supremacy in terms of the number of articles it contributes, but it also maintains a high number of direct relationships with 32 countries, thus accounting for its centrality within the group of countries covered in the graph. Additionally, the country-based analysis displays a higher concentration in terms of the country authorship connections.

**Figure 5:**
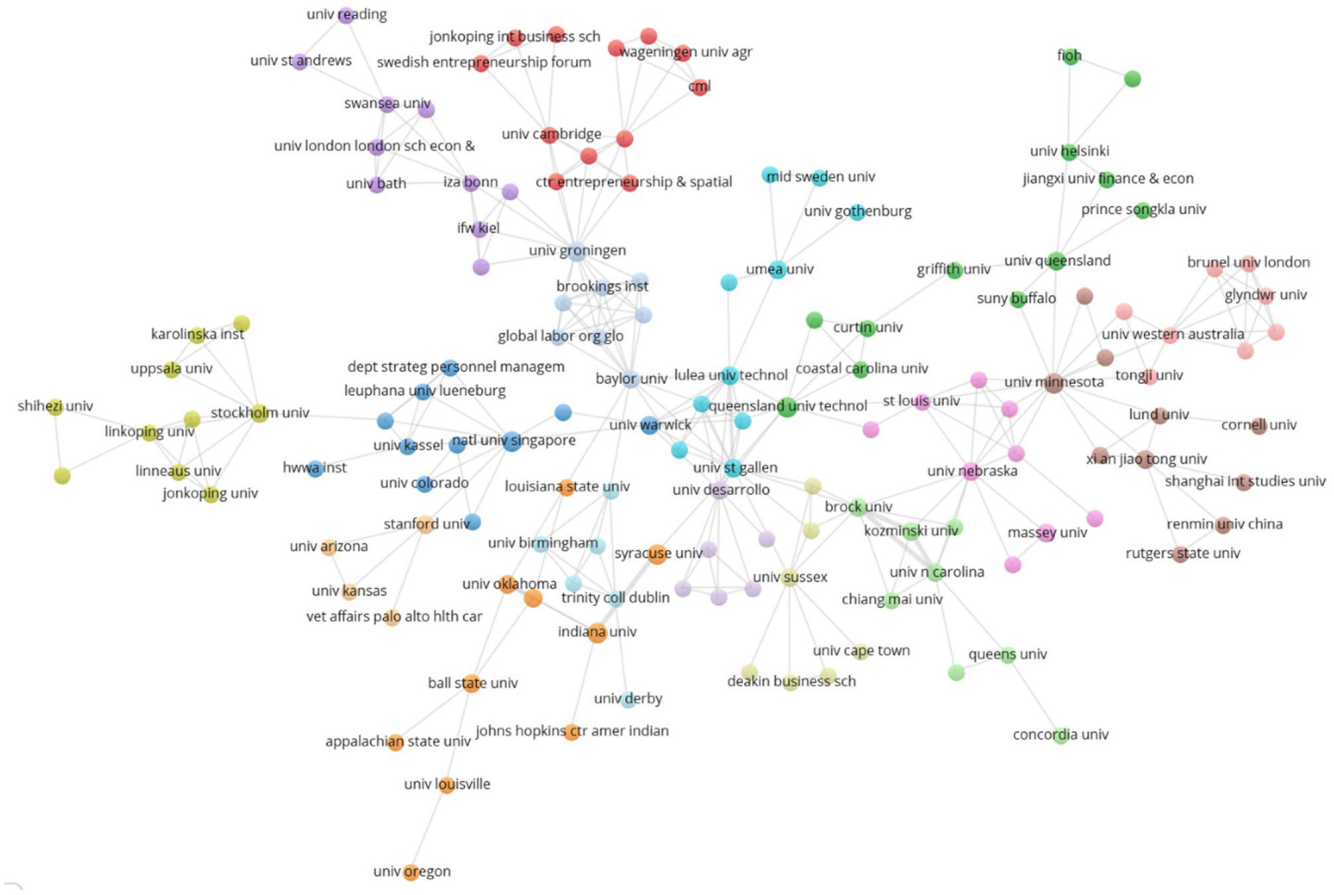
Organizational co-authorship.

This higher level of country-based concentration could better be understood by desegregating the co-authorship level. Figure 5 provides more significant network organizational details. Figure 5 shows that there are 141 consistently connected nodes out of 523 nodes (27.0%). Co-authorship analysis distinguishes 15 clusters that account for groups of reduced size. Those reduced size groups are indirectly linked. All in all, most of the institutions that serve as a bridge between two or more groups, for example, the University of Warwick, Baylor University, and, to a lesser extent, Stockholm University and the University of Groningen, and these enact a high power of intermediation.

**Figure 6:**
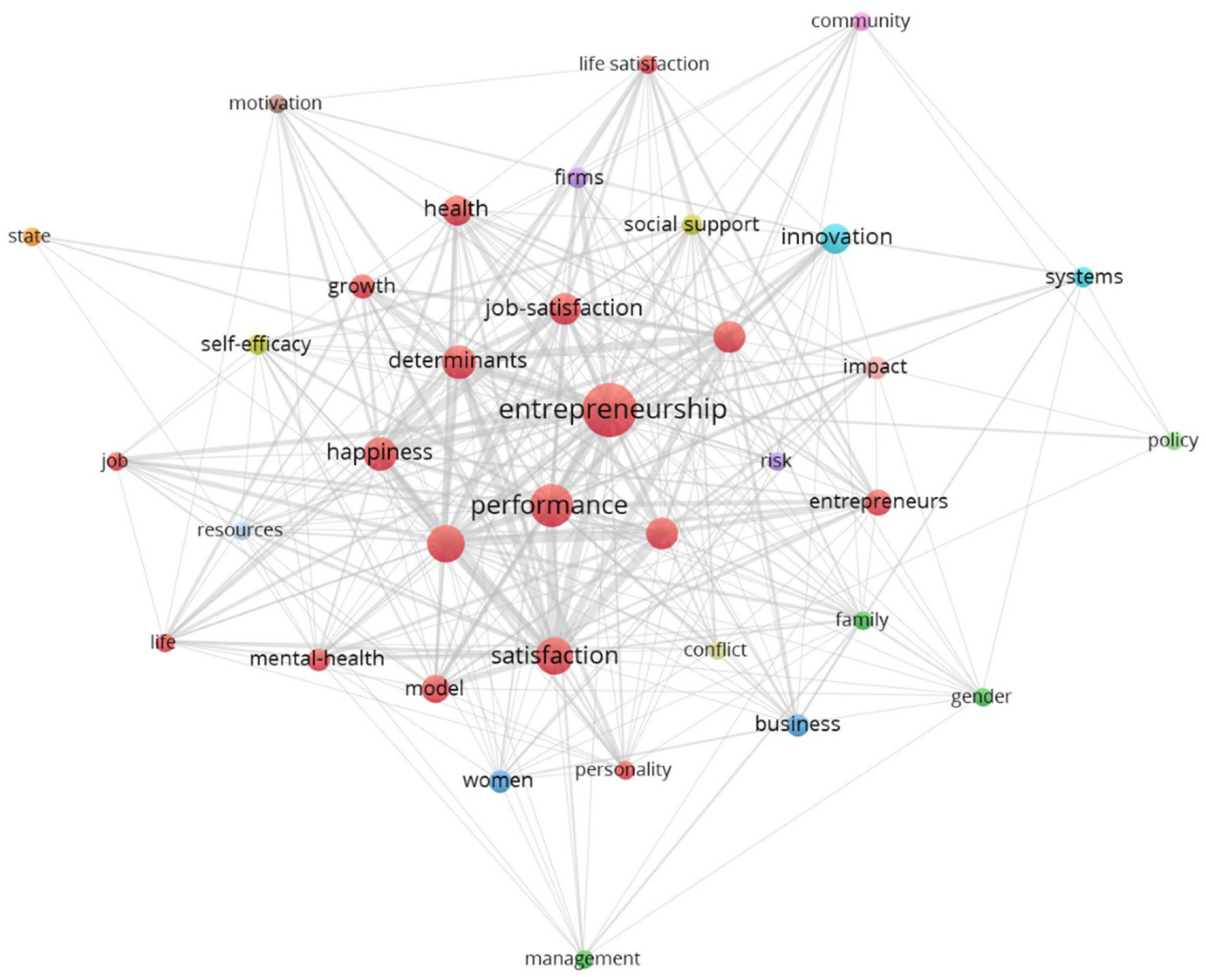
Outstanding Key Word Plus-KWP.

Clarivate analytics established the so-called Keyword Plus® -KWP. KWP are 975 words that Clarivate presented as metadata for the 331 articles in this study dataset. Furthermore, using Zipf's Law, the authors found there were 36 relevant words. Zipf's Law was calculated using the square root of 975 KWP. In the final analysis, this analysis considered 36 KWP with an occurrence number equal to or >8, see Figure 6 [and details in Appendix A (Supplementary Material)]. For a detailed analysis, see Appendix A in the Supplementary Material. This analysis tried represent thematic areas in detail using Outstanding Keyword Plus—OKWP. Fifteen clusters covered thematic areas of research institutions. Further, clusters presented coverage of the relevant topics with variations from 4 to 23. These inter-cluster variations are near related to its paper composition. Each cluster presents a range that goes from 2 to 15 articles.

**Figure 7:**
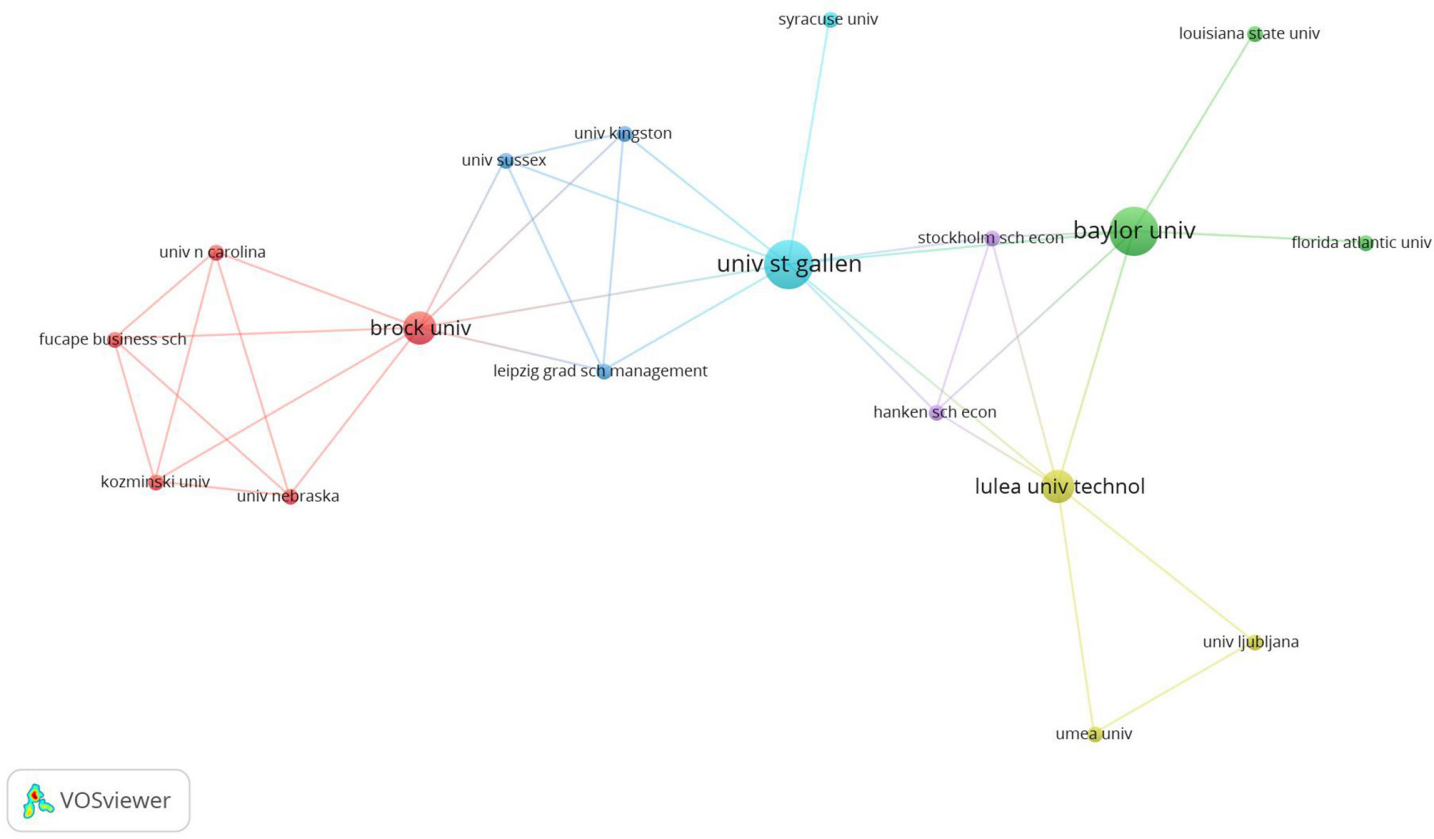
Intermediary organizations clusters.

The intersection between clusters of institutions and the OKWP lead to the identification of 86 articles that shape the structural network of knowledge about entrepreneurial wellbeing knowledge production. In the set mentioned above, a reduced number of 17 articles within 331 are of vital importance (see Appendix B in the Supplementary Material). Those articles contain the OKWPs among their metadata, and, additionally, make it possible to identify the intermediary institutions that make the connection between two or more clusters possible. Furthermore, Figure 7 represents the co-authorship connections between researchers from 18 universities. Among these universities, the following stand out: the University of St. Gallen (Switzerland), Baylor University (Texas, US), Brock University (Ontario, Canada), and Luleå University of Technology (Sweden). Those higher education institutions stand out for their outstanding contribution to the subgroup social cohesion in the global epistemic community that addresses the bi-univocal effects between Wellbeing and Entrepreneurship (Burt, 1987, 2009; Knoke and Laumann, 2012). In particular, that the research structure of the tension between entrepreneurship and wellbeing is articulated with the presence of Swiss, Swedish, and Canadian business schools, countries located among the 10 most sustainable states in the world (Andrejuk, 2019; Ziaja et al., 2019; The Fund for Peace, 2021), can set a trend for this study topic by approaching business from a perspective conditioned to another social context.

**Figure 8:**
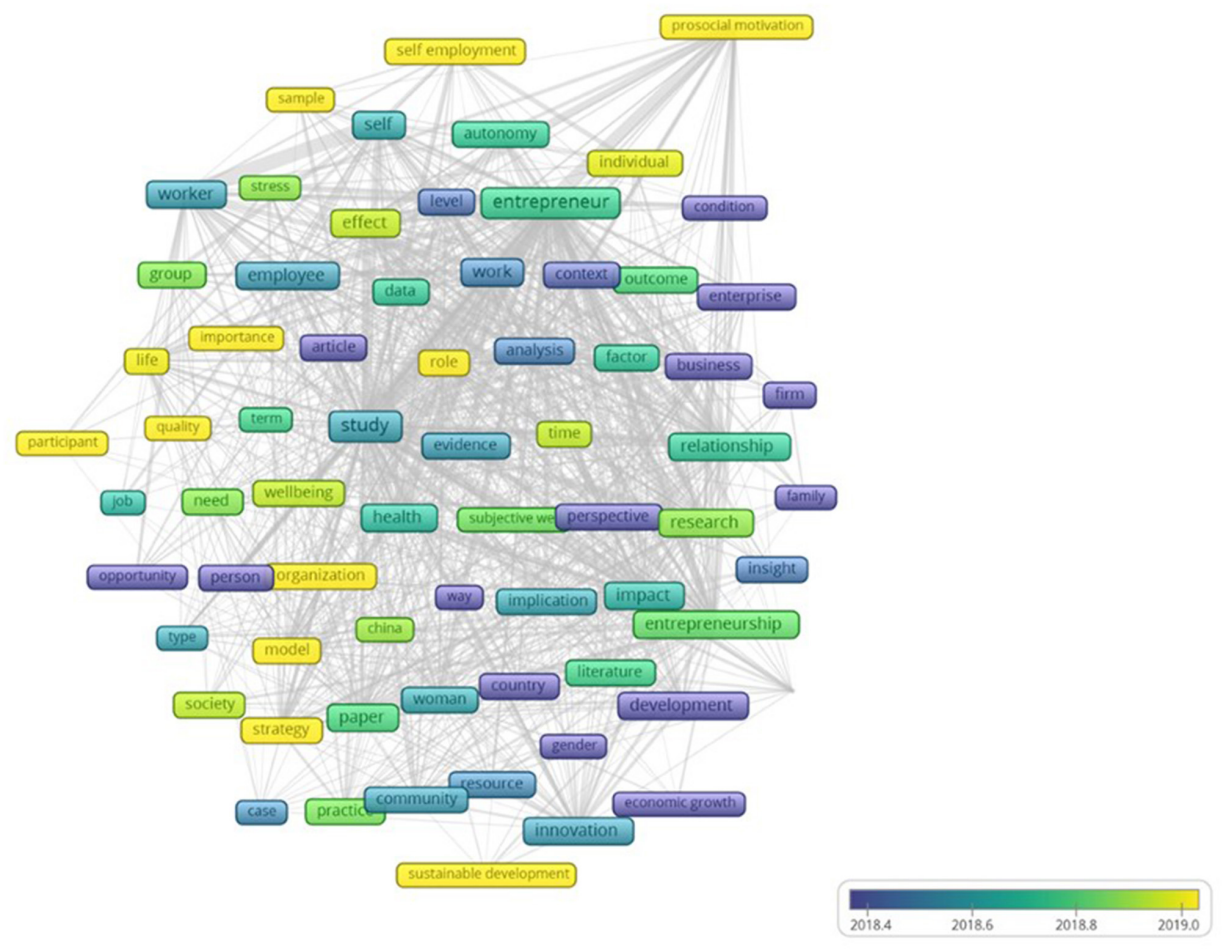
Key terms.

Finally, the authors have carried out an analysis of the corpus made up of the titles and abstracts of this study dataset. That is the last step to understanding the knowledge production in the field of entrepreneurial wellbeing. To perform the analysis, the authors used the VOSviewer tool with 159 articles out of 331 found in the contemporary semi-period 2017–2020 of publications. The analysis mentioned above yielded a total of 4,950 terms. By applying Zipf's Law [square_root (4,950) = 70], we reduced these 4,950 terms to 70 key terms. Figure 8 coincides with an occurrence or repetition of each concept >15 times in the corpus (see Appendix C in the Supplementary Material).

The corpus analysis shows that there are some strictly methodological concepts [e.g., analysis (69), article (43), case (24), context (57), data (41), effect (81), study (227)] being used. Further textual analysis offers some moderating variables terms [e.g., gender (16)] and effects in economic-business terms [e.g., development (71), business (68), strategy (46), economic growth (16), self-employment (36), and social enterprise (35)]. Finally, the use of textual analysis tools revealed psychosocial effect terms in the article database [e.g., wellbeing (58), autonomy (25), community (54), individual (30), family (22), prosocial motivation (18), and stress (25)]. Plus, it is relevant to point out several mentions specific to China (17), that is, the only country that stands out within the metadata set. All in all, from the whole group of terms that the corpus offers, the graph (see Figure 8) only recognizes the term strategy—between the economic-business terms and self-employment and motivation prosocial—as a current psychosocial trend. Regarding these trend terms and the documents that used them, through the analysis of 159 articles, we found a longitudinal study in the UK that relates household self-employment and gender, finding that women appreciate more labor flexibility, being able to combine self-employment with the family in a better way than men (Reuschke, 2019). Furthermore, there is another study that relates to self-employed from China, Russia, and Ukraine. This second study finds that women from Ukraine and Russia have lower rates of self-employment than men, highlighting their propensity for salaried work, while in China, labor rates are much lower both in self-employment and in jobs (Pham et al., 2018).

## Discussion and Conclusions

Entrepreneurship is a fast-growing global phenomenon (Bosma, 2013). This study demonstrates that, in recent years, there has been an exponential growth in the interest of studying entrepreneurs from a psychosocial-eudaimonic approach. Established in the literature, entrepreneurship is a process phenomenon where actors enmesh goals, desires, and hopes with their real-life action. Consequently, entrepreneurship may facilitate the fulfillment of a person's fundamental psychological needs, and it is a critical aspect that affects, for good or for bad, psychological wellbeing (Williams and Shepherd, 2016; Shepherd and Patzelt, 2017; Shir et al., 2019). Following Wiklund et al. (2019) “the experience of satisfaction, positive affect, infrequent negative affect, and psychological functioning in relation to developing, starting, growing, and running an entrepreneurial venture” definition of entrepreneurial wellbeing; this systematic review has delved deeper into the interesting relationship between entrepreneurship and wellbeing.

To track the relationship between entrepreneurship and wellbeing, the study offers a contextual review that leads toward a grounded scientometric systematic analysis of wellbeing and entrepreneurship. Wellbeing and entrepreneurship literature needs to be open to critique and dispute. With a strong scientometric and systematic review of many well-selected articles, the present study contributes to improving the understanding of the link between entrepreneurship and wellbeing knowledge production in terms of job satisfaction and wellbeing, entrepreneurship and self-efficacy, entrepreneurship and health, and entrepreneurship and life satisfaction.

Compared to the results of Sánchez-García et al. (2018), this research offers an upgrade, not just in terms of the recent literature development and discussions but also, and maybe more importantly, in terms of database and search criteria. Therefore, the contribution of this research is two-fold. First, in terms of methodology, the use of a more robust approach to search for the scientometric trends about entrepreneurship wellbeing. Secondly, the present research updates the search for results for the last 2 years of knowledge production, incorporating the inclination to entrepreneurship as a source of hierarchical autonomy and the incorporation of prosocial behaviors (Marín, 2010). This upgrade is particularly important in a research field that presents exponential growth, where 2019 and 2020 present almost double the knowledge production of 2017 and 2018. All in all, with a more grounded search strategy and the update of the scientometric results, this article intended to answer the following research questions: What is the nature of the evolution of scientific knowledge in the entrepreneurial wellbeing field? What is the nature of the concentration in terms of geographical distribution and co-authorship level of knowledge production in the entrepreneurial wellbeing field? What are the knowledge trends in knowledge production for the entrepreneurial wellbeing literature?

In terms of the following question, “What is the nature of the evolution of scientific knowledge in the entrepreneurial wellbeing field?”, results of this study demonstrated that the field of entrepreneurship wellbeing presents an exponential knowledge production growth process. The 331 articles indexed at WoS-JCR on the topic of entrepreneurship and wellbeing that are part of this study database are still not concentrated in any academic journal. However, they are highly concentrated in the US, United Kingdom, and Germany.

The higher level of concentration in terms of geographical zones (Figure 4) correlates with the results about the question on the co-authorship level of knowledge production in the entrepreneurial wellbeing field. Co-authorship analysis leads to finding 15 clusters that account for groups of reduced size (Figure 5). In these networks of co-authorships, there are institutions that concentrate a high power based on their intermediation between institutional networks. In a research field that presents an exponential knowledge production growth process, intermediation offers the opportunity to position the institution getting the opportunities of structural holes (Burt, 2004) in this novel field. Furthermore, those actors that intermediate in the co-authorship networks, as is the case for the University of St. Gallen (Switzerland) and Baylor University (Texas, US), stand out for their outstanding contribution to the social cohesion in the global knowledge production community researching wellbeing and entrepreneurship.

Regarding the trends about knowledge production in the entrepreneurial wellbeing literature, the research presents a topics series intricately connected to the background literature offered in this article. The background literature review offers topics such as entrepreneurship and its relationships with life satisfaction, health, self-efficacy, happiness, and job satisfaction. These topics are highly correlated with the scientometric results of the present study. Entrepreneurship wellbeing, i.e., the feeling of satisfaction related to creating, opening, expanding, and managing an entrepreneurial endeavor, is a research field that presents a thematic continuity since 1995. Those themes, which are represented by the Keyword Plus® at the database, are at the core of the knowledge production trends of this epistemic community. However, it is only by studying the intersection between institutions and keyword plus clusters that the structural pattern appears. In fact, we analyzed the keyword plus network, from Clarivate, and clusters based on the 331-article database of this study, and Figure 7 shows that happiness, satisfaction, job satisfaction, and health are highly displayed in the network of institutions and keywords. These structural aspects of the research field show new avenues about entrepreneurship wellbeing presented by Sánchez-García et al. (2018).

As a conclusion, this research invites scholars in entrepreneurship and wellbeing to continue their exploration on topics such as public policies that promote the wellbeing of entrepreneurial activity; studies of the effects of wellbeing in the generation of wealth; promotion models based on wellbeing-based ventures; ecosystems of wellness ventures; and productive development and entrepreneurship of local and community wellbeing. Those themes are less represented within the corpus of the systematically analyzed literature and could offer a tremendous opportunity to those scholars that are researching the effects of entrepreneurship work, and it is affected by feelings of happiness, satisfaction, job-satisfaction, and health.

In terms of implications to practitioners and to business more broadly, the present article leads the inquiry toward deeper subjective wellbeing and its relationship with the entrepreneurship practice and psycho-social context that impacts labor market relationships (Sridharan et al., 2014; Liang and Goetz, 2016; Bernhard-Oettel et al., 2019; Burke and Cowling, 2020; Gevaert et al., 2020). This study also invites them to focus on the adoption of a new lens to business creation that is based on the business thinking of latitudes with much greater social stability (Welsh et al., 2016; Kibler et al., 2019; Shir et al., 2019). Decision-makers at the government and corporation levels must be aware of new insights that appear in this stream of literature, which deepens our understanding of these issues (Hmieleski and Sheppard, 2019; Nordenmark et al., 2019; Giraldo et al., 2020; Holm et al., 2020; Kluczewska, 2020; Xu et al., 2020). This is of particular importance in pandemic times where the people's mental health and wellbeing are being called for each corporate and business operation (Carnevale and Hatak, 2020).

## Limitations

This study has some limitations. Firstly, given the breadth of the works reviewed in this article, we can lay the foundations for the expansion of studies that relate entrepreneurship to wellbeing, which will be required in the future. But focusing this systematic review on SSCI + SCIE databases, only considering articles that are part of JCR-WoS journals collection, creates a limitation in the scope of the sample to avoid adding irrelevant articles to the study dataset. A trade-off for having a significant impact worldwide (Carabantes-Alarcón and Alou-Cervera, 2019; Serrano et al., 2019) is to assume this scope limitation. Additionally, a strong future methodological challenge is to achieve greater integration between Scientometrics and the eligibility techniques incorporated in PRISMA (PICOS or SPIDER).

Secondly, we should delve into specific application fields, such as Entrepreneurship and job satisfaction, Entrepreneurship and self-efficacy, Entrepreneurship and health, Entrepreneurship and happiness, and Entrepreneurship and life satisfaction. This, as the corpus of articles continues to grow exponentially over time, can be improved as there is a critical mass of research in each of these topics.

Thirdly, this study details thematic trends but does not analyze the academic trajectory of prolific authors, although it identifies common patterns that can be of significant interest in the training of future young researchers.

Fourth, this review is mainly limited to a study that descriptive about the knowledge production between the intersection of wellbeing and entrepreneurship topics, establishing relevance, concentrations, and relationships between various data and metadata that characterize the articles selected as the corpus studied.

Finally, the expected changes in the business conception that the global pandemic from Sars-Cov-2 has imposed on us (Carnevale and Hatak, 2020; Saiz-Álvarez et al., 2020; Ahmad et al., 2021) could generate changes in this interrelation, increasing the tension between entrepreneurship and wellbeing and creating forms of defense against the negative effects (Hernández-Sánchez et al., 2020). This is a phenomenon that should be studied in a “New Normality” stage.

## Data Availability Statement

The original contributions presented in the study are included in the article/Supplementary Material, further inquiries can be directed to the corresponding author/s.

## Author Contributions

AV-M structured and extracted most of the information and produced a draft of the results. NC-B extracted additional information and added it to the analysis. NC-B and GS-S reviewed the literature to produce the conceptual background of the study. AA-M, JE-C, and AV-M drafted several parts of the article and, together with AV-M and NC-B, analyzed the data set. All of the authors are fully responsible for the totality of the work and followed a strict ethical and integrity protocol to produce and present the results of their research. All authors conceptualized this scientometrics systematic review.

## Conflict of Interest

The authors declare that the research was conducted in the absence of any commercial or financial relationships that could be construed as a potential conflict of interest.
