# Supplementary material for: A Scientometric Systematic Review of Entrepreneurial Wellbeing Knowledge Production
Source: Front Psychol. 2021 Mar 31;12:641465. doi: 10.3389/fpsyg.2021.641465 (PMC8044348; doi:10.3389/fpsyg.2021.641465)
Supplement: Supplementary file 1 [file Data_Sheet_1.docx]

# Supplementary Material

Appendix A

**Table 1 Co-authorship level clusters**

| # Cluster (color/items) | Organizations | # Articles | OKWP |
| --- | --- | --- | --- |
| 1 (light purple /16) | Aalto Univ, Coastal Carolina Univ, Curtin Univ, Hanken Sch Econ, IZA Inst study labor, Lulea Univ Technol, Queensland Univ Technol, Stockholm Sch Econ, United Nations Univ UNU MERIT, Univ Alcala de Henares, Univ Desarrollo, Univ Maastricht, Univ San Sebastian, Univ St Gallen, Univ Warwick, Yonsei Univ. | 15 | 22 KWP: entrepreneurship, performance, satisfaction, work, determinants, happiness, job-satisfaction, self-employment, stress, model, entrepreneurs, growth, mental health, business, self-efficacy, social support, conflict, life, life satisfaction, motivation, job. |
| 2 (light green /15) | Brock Univ, Chiang Mai Univ, Concordia Univ, Deakin Business Sch, Fucape Business Sch, Kozminski Univ, Leipzig Grad Sch Management, Max Planck Inst Econ, Queens Univ, Univ Cape Town, Univ Kingston, Univ N Carolina, Univ Sussex, Univ Waterloo, Warwick Business Sch | 12 | 23 KWP: entrepreneurship, performance, satisfaction work, determinants, happiness, job-satisfaction, self-employment, stress, innovation, model, business, firms, self-efficacy, social support, systems, family, community, gender, job, policy, resources. |
| 3 (red /12) | CML, Ctr Entrepreneurship & Spatial Econ, Jonkoping Int Business Sch, KTH Royal Inst Technol, NHL Stenden Univ Appl Sci, Plant Protect Serv, Plant Res Int, Res Inst Ind Econ IFN, Swedish Entrepreneurship Forum, Univ Cambridge, Wageningen Univ, Wageningen Univ Agr. | 3 | 6 KWP: entrepreneurship, determinants, happiness, self-employment, systems, personality. |
| 4 (pink /12) | Illinois State Univ, Iowa State Univ, Kansas State Univ, Massey Univ, Mastercard FDN, St Louis Univ, UNICEF, Univ Leipzig, Univ Minnesota, Univ Nebraska, Univ Tennessee, Univ Waikato. | 13 | 17 KWP: entrepreneurship, performance, satisfaction, work, determinants, job-satisfaction, self-employment, stress,  health, innovation, entrepreneurs, growth, impact, firms, social support, life satisfaction, resources. |
| 5 (orange/10) | Appalachian State Univ, Ball State Univ, Indiana Univ, Johns Hopkins Ctr Amer Indian Hlth, Louisiana State Univ, Syracuse Univ, Univ Louisville, Univ Oklahoma, Univ Oregon, Villanova Univ. | 13 | 17 KWP: entrepreneurship, performance, satisfaction, work, self-employment, health, innovation, entrepreneurs, growth, firms, self-efficacy, social support, family, motivation, community, job, risk. |
| 6 (blue /10) | Dept Strateg Personnel Management, HWWA Inst, Johannes Gutenberg Univ Mainz, Leuphana Univ Lueneburg, Leuphana Univ Luneburg, Nanyang Technol Univ, Natl Univ Singapore, Tulane Univ, Univ Colorado, Univ Kassel. | 7 | 14 KWP: entrepreneurship, performance, satisfaction, work, job-satisfaction,  health, model, mental health, self-efficacy, social support, life, job, management, resources. |
| 7 (golden /10) | Jonkoping Univ, Karolinska Inst, Linkoping Univ, Linneaus Univ, Malardalen Univ, Shihezi Univ, Stockholm Univ, Swedish meteorol & Hydrol Inst, Swedish Univ Agr Sci. | 5 | 12 KWP: entrepreneurship, satisfaction, stress, health, entrepreneurs, mental health, impact, social support, gender, management, policy, resources. |
| 8 (purple /10) | Aberystwyth Univ, Friedrich Schiller Univ Jena, IFW Kiel, IZA Bonn, John Cabot Univ Rome, Swansea Univ, Univ Bath, Univ London London Sch Econ & Polit Sci, Univ Reading, Univ St Andrews. | 3 | 8 KWP: entrepreneurship, satisfaction, determinants, job-satisfaction, self-employment, growth, life, personality. |
| 9 (light cerise /8) | Brunel Univ London, Coventry Univ, Glyndwr Univ, ICFAI FDN Higher Educ, Tongji Univ, Univ Elect Sci & Technol China, Univ Lincoln, Univ Western Australia. | 2 | 4 KWP: entrepreneurship, performance, innovation, social support.  4 OKWP: entrepreneurship, performance, innovation, social support. |
| 10 (green /8) | FIOH, Griffith Univ, Jiangxi Univ Finance & Econ, Nat Resources Inst Finland Luke, Prince Songkla Univ, Suny Buffalo, Univ Helsinki, Univ Queensland. | 7 | 6 KWP: work, determinants, health, model, community, gender. |
| 11 (light blue /8) | Baylor Univ, Brookings Inst, CERGE, Global Labor Org GLO, Inst Labor Econ IZA, Leibniz Inst East & Southeast European Studies IO, Univ Groningen, Ural Fed Univ. | 8 | 18 KWP: entrepreneurship, satisfaction, work, determinants, happiness, job-satisfaction, self-employment, health, entrepreneurs, mental health, business, women, firms, family, life, life satisfaction, motivation, community. |
| 12 (brown /7) | Cornell Univ, Lund Univ, Renmin Univ China, Rutgers State Univ, Shanghai Int Studies Univ, Univ Chicago, Xi an Jiao Tong Univ. | 5 | 9 KWP: entrepreneurship, performance, job-satisfaction, self-employment, entrepreneurs, business, women, firms, risk. |
| 13 (light sky blue /6) | Florida Atlantic Univ, Trinity Coll Dublin, Univ Birmingham, Univ Derby, Univ Minneapolis, Western Univ. | 3 | 4 KWP: performance, happiness, job-satisfaction, health. |
| 14 (sky blue /5) | Karlstad Unv, Mid Sweden Univ, UMEA Univ, Univ Gothenburg, Univ Ljubljana. | 4 | 13 KWP: entrepreneurship, satisfaction, work, determinants, happiness, stress, entrepreneurs, growth, mental health, family, life, job, personality. |
| 15 (light orange/4) | Stanford Univ, Univ Arizona, Univ Kansas, Vet Affairs Palo Alto Hlth Care Syst | 4 | 6 KWP: satisfaction, health, impact, life, personality, policy. |

**Outstanding Keyword Plus (occurrences):** entrepreneurship (52), performance (36), satisfaction (28), work (28), determinants (24), happiness (22), job-satisfaction (20), self-employment (20), stress (20), health (19), innovation (19), model (16), entrepreneurs (14), growth (13), mental health (12), business (11), impact (11), women (11), firms (10), self-efficacy (10), social support (10), systems (10), conflict (9), family (9), life (9), life satisfaction (9), motivation (9), community (8), gender (8), job (8), management (8), personality (8), policy (8), resources (8), risk (8), state (8).

 Appendix B

**Table 2 Intersection between institutions cluster through keyword plus**

| # | Intersections between cluster | Article (UT) |
| --- | --- | --- |
|  | 1, 4 | WOS:000392357900002 |
|  | 4, 10 | WOS:000330726000007 |
|  | 4, 12 | WOS:000432768600002 |
|  | 5, 11 | WOS:000301472000010 |
|  | 1, 11 | WOS:000484653600002 |
|  | 1, 14 | WOS:000285690800001 |
|  | 2, 4 | WOS:000431871000007 |
|  | 1, 5 | WOS:000541934200015 |
|  | 11, 13 | WOS:000524349700008 |
|  | 3, 11 | WOS:000470948800002 |
|  | 1, 10 | WOS:000467884300007 |
|  | 1, 2, 6 | WOS:000455502100003 |
|  | 8, 11 | WOS:000496892700005 |
|  | 4, 9 | WOS:000478329600001 |
|  | 6, 7 | WOS:000415994400006 |
|  | 1, 2 | WOS:000529843800001 |
|  | 6, 15 | WOS:000503130000001 |

Search vector for 17 bridge articles between clusters:

UT=(WOS:000392357900002 OR WOS:000330726000007 OR WOS:000432768600002 OR WOS:000301472000010 OR WOS:000484653600002 OR WOS:000285690800001 OR WOS:000431871000007 OR WOS:000541934200015 OR WOS:000524349700008 OR WOS:000470948800002 OR WOS:000467884300007 OR WOS:000455502100003 OR WOS:000496892700005 OR WOS:000478329600001 OR WOS:000415994400006 OR WOS:000529843800001 OR WOS:000503130000001)

**Appendix C:** Table 3 VOSviewer Output Text Data Key Terms (Alphabetic order)

id term occurrences relevance score

1 analysis 69 0.1914

2 article 43 0.3426

3 autonomy 25 1.5248

4 business 68 0.3051

5 case 24 1.0843

6 challenge 34 0.3088

7 china 17 0.4528

8 community 54 1.2062

9 condition 23 1.7078

10 context 57 0.8066

11 country 45 0.7141

12 data 41 0.219

13 development 71 0.8885

14 economic growth 16 2.2019

15 effect 81 0.9978

16 employee 66 1.0901

17 enterprise 41 0.3464

18 entrepreneur 196 0.5941

19 entrepreneurship 136 0.6865

20 evidence 36 0.5607

21 factor 50 0.3673

22 family 22 1.0934

23 firm 34 0.9147

24 gender 16 1.1537

25 group 36 2.8915

26 health 73 0.539

27 impact 89 0.4908

28 implication 49 0.1919

29 importance 23 0.1356

30 individual 30 3.0497

31 innovation 77 1.3742

32 insight 30 0.3904

33 job 21 1.1542

34 level 53 0.488

35 life 51 0.4305

36 literature 46 0.5514

37 model 62 0.2226

38 need 37 0.3231

39 opportunity 29 1.9632

40 organization 31 0.506

41 outcome 48 0.0709

42 paper 76 0.3324

43 participant 19 1.0638

44 person 54 0.4672

45 perspective 43 0.1956

46 practice 47 1.0253

47 prosocial motivation 18 4.2265

48 quality 29 0.6677

49 relationship 106 0.1626

50 research 82 0.3017

51 resource 33 0.8594

52 role 61 0.1323

53 sample 29 1.2314

54 self 78 4.0214

55 self employment 36 3.9128

56 social enterprise 35 2.2875

57 society 29 0.5506

58 strategy 44 1.1457

59 stress 25 2.329

60 study 227 0.1169

61 subjective well 22 0.381

62 sustainable development 17 2.5537

63 term 28 0.2412

64 time 31 0.284

65 type 23 0.3086

66 way 26 0.6362

67 well-being 58 0.4961

68 woman 61 0.9593

69 work 91 0.4195

70 worker 67 4.159
